# Supplementary material for: Effects of media multitasking frequency on a novel volitional multitasking paradigm
Source: PeerJ. 2022 Jan 27;10:e12603. doi: 10.7717/peerj.12603 (PMC8801180; doi:10.7717/peerj.12603)
Supplement: Supplemental Information 1 — mi denotes the number of media types used in conjunction with the primary medium, i, corresponds to the number of hours spent using the primary medium, and htotal is the total amount of hours spent using all primary media types. Equation used according to Ophir, Nass & Wagner (2009). Briefly, the index is calculated by assigning numeric values to each of the matrix answers and weighing the sum of these values across each primary medium by the percentage of time spent with the corresponding primary medium. [file peerj-10-12603-s001.pdf]

$$\text{MMI} = \sum_{i=1}^{11} \frac{m_i \times h_i}{h_{\text{total}}}$$
