# Supplementary material for: Effects of media multitasking frequency on a novel volitional multitasking paradigm
Source: PeerJ. 2022 Jan 27;10:e12603. doi: 10.7717/peerj.12603 (PMC8801180; doi:10.7717/peerj.12603)
Supplement: Supplemental Information 2 — Note. M and SD are used to represent mean and standard deviation, respectively. Values in square brackets indicate the 95% confidence interval for each correlation. The confidence interval is a plausible range of population correlations that could have caused the sample correlation (Cumming, 2014). * indicates p < .05. ** indicates p < .01. [file peerj-10-12603-s002.docx]

Supplemental Table S1

*Means, standard deviations, and correlations with confidence intervals for all surveys and behavioral measures*

| Variable | *M* | *SD* | 1 | 2 | 3 | 4 | 5 | 6 | 7 | 8 | 9 | 10 | 11 | 12 |
| --- | --- | --- | --- | --- | --- | --- | --- | --- | --- | --- | --- | --- | --- | --- |
|  |  |  |  |  |  |  |  |  |  |  |  |  |  |  |
| 1. MMI Score | 2.95 | 1.28 |  |  |  |  |  |  |  |  |  |  |  |  |
|  |  |  |  |  |  |  |  |  |  |  |  |  |  |  |
| 2. MPI Score | 38.54 | 10.93 | -.10 |  |  |  |  |  |  |  |  |  |  |  |
|  |  |  | [-.30, .11] |  |  |  |  |  |  |  |  |  |  |  |
|  |  |  |  |  |  |  |  |  |  |  |  |  |  |  |
| 3. BIS Attentional | 17.76 | 3.86 | .09 | .29** |  |  |  |  |  |  |  |  |  |  |
|  |  |  | [-.12, .29] | [.09, .47] |  |  |  |  |  |  |  |  |  |  |
|  |  |  |  |  |  |  |  |  |  |  |  |  |  |  |
| 4. Switch Rate | 0.31 | 0.30 | -.08 | .40** | .13 |  |  |  |  |  |  |  |  |  |
|  |  |  | [-.28, .13] | [.21, .56] | [-.08, .32] |  |  |  |  |  |  |  |  |  |
|  |  |  |  |  |  |  |  |  |  |  |  |  |  |  |
| 5. PopupSelect | 1.31 | 0.35 | -.12 | -.12 | -.15 | -.21 |  |  |  |  |  |  |  |  |
|  |  |  | [-.35, .12] | [-.35, .12] | [-.38, .09] | [-.43, .03] |  |  |  |  |  |  |  |  |
|  |  |  |  |  |  |  |  |  |  |  |  |  |  |  |
| 6. Return Cost | 0.34 | 0.41 | .18 | -.18 | -.08 | -.38** | .31* |  |  |  |  |  |  |  |
|  |  |  | [-.07, .41] | [-.41, .07] | [-.32, .17] | [-.57, -.15] | [.08, .52] |  |  |  |  |  |  |  |
|  |  |  |  |  |  |  |  |  |  |  |  |  |  |  |
| 7. Interference Cost | 0.01 | 0.22 | .08 | .10 | -.03 | -.11 | .01 | -.01 |  |  |  |  |  |  |
|  |  |  | [-.14, .28] | [-.11, .31] | [-.24, .18] | [-.31, .10] | [-.23, .26] | [-.26, .23] |  |  |  |  |  |  |
|  |  |  |  |  |  |  |  |  |  |  |  |  |  |  |
| 8. PrimaryRepeat | 2.30 | 0.41 | .22* | .19 | .08 | .07 | .15 | .36** | -.04 |  |  |  |  |  |
|  |  |  | [.02, .41] | [-.01, .39] | [-.13, .28] | [-.14, .27] | [-.09, .38] | [.13, .56] | [-.25, .17] |  |  |  |  |  |
|  |  |  |  |  |  |  |  |  |  |  |  |  |  |  |
| 9. Primary RT | 2.32 | 0.41 | .24* | .20 | .10 | .09 | .14 | .38** | -.04 | 1.00** |  |  |  |  |
|  |  |  | [.03, .42] | [-.01, .39] | [-.11, .30] | [-.12, .29] | [-.10, .37] | [.16, .57] | [-.25, .17] | [1.00, 1.00] |  |  |  |  |
|  |  |  |  |  |  |  |  |  |  |  |  |  |  |  |
| 10. Secondary RT | 2.62 | 0.50 | .09 | -.07 | .01 | -.03 | -.11 | .38** | .13 | .29* | .30* |  |  |  |
|  |  |  | [-.15, .33] | [-.31, .18] | [-.24, .25] | [-.27, .22] | [-.35, .14] | [.15, .58] | [-.12, .37] | [.05, .50] | [.06, .51] |  |  |  |
|  |  |  |  |  |  |  |  |  |  |  |  |  |  |  |
| 11. PrimaryNopopup | 2.31 | 0.42 | .24* | .19 | .10 | .09 | .14 | .39** | -.08 | 1.00** | 1.00** | .29* |  |  |
|  |  |  | [.03, .42] | [-.02, .38] | [-.11, .30] | [-.12, .29] | [-.10, .37] | [.16, .58] | [-.29, .13] | [.99, 1.00] | [1.00, 1.00] | [.05, .50] |  |  |
|  |  |  |  |  |  |  |  |  |  |  |  |  |  |  |
| 12. PopupIgnore | 2.33 | 0.46 | .26* | .23* | .08 | .05 | .13 | .34** | .40** | .90** | .90** | .33** | .88** |  |
|  |  |  | [.05, .44] | [.02, .42] | [-.13, .28] | [-.16, .25] | [-.11, .36] | [.11, .54] | [.21, .56] | [.84, .93] | [.85, .93] | [.09, .53] | [.82, .92] |  |
|  |  |  |  |  |  |  |  |  |  |  |  |  |  |  |
| 13. PrimaryReturn | 2.69 | 0.71 | .25* | .01 | -.02 | -.23 | .21 | .82** | -.07 | .83** | .84** | .45** | .84** | .73** |
|  |  |  | [.00, .46] | [-.24, .25] | [-.27, .22] | [-.45, .02] | [-.03, .43] | [.73, .89] | [-.31, .18] | [.73, .89] | [.75, .90] | [.23, .63] | [.75, .90] | [.59, .83] |
|  |  |  |  |  |  |  |  |  |  |  |  |  |  |  |

*Note.* *M* and *SD* are used to represent mean and standard deviation, respectively. Values in square brackets indicate the 95% confidence interval for each correlation. The confidence interval is a plausible range of population correlations that could have caused the sample correlation (Cumming, 2014). * indicates *p* < .05. ** indicates *p* < .01.
