# Supplementary material for: Effects of media multitasking frequency on a novel volitional multitasking paradigm
Source: PeerJ. 2022 Jan 27;10:e12603. doi: 10.7717/peerj.12603 (PMC8801180; doi:10.7717/peerj.12603)
Supplement: Supplemental Information 3 — Note. A significant b-weight indicates the beta-weight and semi-partial correlation are also significant. b represents unstandardized regression weights. beta indicates the standardized regression weights. sr2 represents the semi-partial correlation squared. r represents the zero-order correlation. LL and UL indicate the lower and upper limits of a confidence interval, respectively. * indicates p < .05. ** indicates p < .01. [file peerj-10-12603-s003.docx]

Supplemental Table S2

*Regression results using switch rate as the criterion*

| Predictor | *b* | *b*  95% CI  [LL, UL] | *beta* | *beta*  95% CI  [LL, UL] | *sr^2^* | *sr^2^*  95% CI  [LL, UL] | *r* | Fit | Difference |
| --- | --- | --- | --- | --- | --- | --- | --- | --- | --- |
| (Intercept) | 0.36** | [0.20, 0.52] |  |  |  |  |  |  |  |
| MMI Score | -0.02 | [-0.07, 0.03] | -0.08 | [-0.29, 0.14] | .01 | [.00, .07] | -.08 |  |  |
|  |  |  |  |  |  |  |  | *R^2^*  = .006 |  |
|  |  |  |  |  |  |  |  | 95% CI[.00,.07] |  |
|  |  |  |  |  |  |  |  |  |  |
| (Intercept) | 0.24 | [-0.19, 0.66] |  |  |  |  |  |  |  |
| MMI Score | -0.02 | [-0.07, 0.03] | -0.09 | [-0.30, 0.13] | .01 | [-.03, .04] | -.08 |  |  |
| Total BIS | 0.00 | [-0.00, 0.01] | 0.07 | [-0.15, 0.28] | .00 | [-.02, .03] | .05 |  |  |
|  |  |  |  |  |  |  |  | *R^2^*  = .010 | Δ*R^2^*  = .004 |
|  |  |  |  |  |  |  |  | 95% CI[.00,.07] | 95% CI[-.02, .03] |
|  |  |  |  |  |  |  |  |  |  |
| (Intercept) | -0.02 | [-0.43, 0.39] |  |  |  |  |  |  |  |
| MMI Score | -0.01 | [-0.05, 0.04] | -0.02 | [-0.23, 0.18] | .00 | [-.01, .01] | -.08 |  |  |
| Total BIS | -0.00 | [-0.01, 0.00] | -0.05 | [-0.26, 0.16] | .00 | [-.02, .02] | .05 |  |  |
| MPI Score | 0.01** | [0.01, 0.02] | 0.41 | [0.21, 0.62] | .16 | [.02, .29] | .40** |  |  |
|  |  |  |  |  |  |  |  | *R^2^*  = .166** | Δ*R^2^*  = .155** |
|  |  |  |  |  |  |  |  | 95% CI[.03,.28] | 95% CI[.02, .29] |
|  |  |  |  |  |  |  |  |  |  |

*Note.* A significant *b*-weight indicates the beta-weight and semi-partial correlation are also significant. *b* represents unstandardized regression weights. *beta* indicates the standardized regression weights. *sr^2^* represents the semi-partial correlation squared. *r* represents the zero-order correlation. *LL* and *UL* indicate the lower and upper limits of a confidence interval, respectively.
* indicates *p* < .05. ** indicates *p* < .01.
