# Supplementary material for: Effects of media multitasking frequency on a novel volitional multitasking paradigm
Source: PeerJ. 2022 Jan 27;10:e12603. doi: 10.7717/peerj.12603 (PMC8801180; doi:10.7717/peerj.12603)
Supplement: Supplemental Information 4 — Note. A significant b-weight indicates the beta-weight and semi-partial correlation are also significant. b represents unstandardized regression weights. beta indicates the standardized regression weights. sr2 represents the semi-partial correlation squared. r represents the zero-order correlation. LL and UL indicate the lower and upper limits of a confidence interval, respectively. * indicates p < .05. ** indicates p < .01. [file peerj-10-12603-s004.docx]

Supplemental Table S3

*Regression results using return cost as the criterion*

| Predictor | *b* | *b*  95% CI  [LL, UL] | *beta* | *beta*  95% CI  [LL, UL] | *sr^2^* | *sr^2^*  95% CI  [LL, UL] | *r* | Fit | Difference |
| --- | --- | --- | --- | --- | --- | --- | --- | --- | --- |
| (Intercept) | 0.15 | [-0.11, 0.42] |  |  |  |  |  |  |  |
| MMI Score | 0.06 | [-0.02, 0.15] | 0.18 | [-0.07, 0.43] | .03 | [.00, .15] | .18 |  |  |
|  |  |  |  |  |  |  |  | *R^2^*  = .033 |  |
|  |  |  |  |  |  |  |  | 95% CI[.00,.15] |  |
|  |  |  |  |  |  |  |  |  |  |
| (Intercept) | 0.36 | [-0.33, 1.05] |  |  |  |  |  |  |  |
| MMI Score | 0.07 | [-0.02, 0.16] | 0.20 | [-0.06, 0.46] | .04 | [-.05, .13] | .18 |  |  |
| Total BIS | -0.00 | [-0.01, 0.01] | -0.08 | [-0.34, 0.18] | .01 | [-.03, .04] | -.03 |  |  |
|  |  |  |  |  |  |  |  | *R^2^*  = .039 | Δ*R^2^*  = .006 |
|  |  |  |  |  |  |  |  | 95% CI[.00,.15] | 95% CI[-.03, .04] |
|  |  |  |  |  |  |  |  |  |  |
| (Intercept) | 0.49 | [-0.23, 1.22] |  |  |  |  |  |  |  |
| MMI Score | 0.06 | [-0.03, 0.15] | 0.18 | [-0.08, 0.44] | .03 | [-.05, .11] | .18 |  |  |
| Total BIS | -0.00 | [-0.01, 0.01] | -0.04 | [-0.30, 0.23] | .00 | [-.02, .02] | -.03 |  |  |
| MPI Score | -0.01 | [-0.02, 0.00] | -0.16 | [-0.42, 0.10] | .02 | [-.05, .09] | -.18 |  |  |
|  |  |  |  |  |  |  |  | *R^2^*  = .062 | Δ*R^2^*  = .023 |
|  |  |  |  |  |  |  |  | 95% CI[.00,.17] | 95% CI[-.05, .09] |
|  |  |  |  |  |  |  |  |  |  |

*Note.* A significant *b*-weight indicates the beta-weight and semi-partial correlation are also significant. *b* represents unstandardized regression weights. *beta* indicates the standardized regression weights. *sr^2^* represents the semi-partial correlation squared. *r* represents the zero-order correlation. *LL* and *UL* indicate the lower and upper limits of a confidence interval, respectively.
* indicates *p* < .05. ** indicates *p* < .01.
