# Supplementary material for: Effects of media multitasking frequency on a novel volitional multitasking paradigm
Source: PeerJ. 2022 Jan 27;10:e12603. doi: 10.7717/peerj.12603 (PMC8801180; doi:10.7717/peerj.12603)
Supplement: Supplemental Information 5 — Note. A significant b-weight indicates the beta-weight and semi-partial correlation are also significant. b represents unstandardized regression weights. beta indicates the standardized regression weights. sr2 represents the semi-partial correlation squared. r represents the zero-order correlation. LL and UL indicate the lower and upper limits of a confidence interval, respectively. * indicates p < .05. ** indicates p < .01. [file peerj-10-12603-s005.docx]

Supplemental Table S4

*Regression results using interference cost as the criterion*

| Predictor | *b* | *b*  95% CI  [LL, UL] | *beta* | *beta*  95% CI  [LL, UL] | *sr^2^* | *sr^2^*  95% CI  [LL, UL] | *r* | Fit | Difference |
| --- | --- | --- | --- | --- | --- | --- | --- | --- | --- |
| (Intercept) | -0.03 | [-0.14, 0.09] |  |  |  |  |  |  |  |
| MMI Score | 0.01 | [-0.02, 0.05] | 0.08 | [-0.14, 0.29] | .01 | [.00, .07] | .08 |  |  |
|  |  |  |  |  |  |  |  | *R^2^*  = .006 |  |
|  |  |  |  |  |  |  |  | 95% CI[.00,.07] |  |
|  |  |  |  |  |  |  |  |  |  |
| (Intercept) | -0.02 | [-0.32, 0.29] |  |  |  |  |  |  |  |
| MMI Score | 0.01 | [-0.02, 0.05] | 0.08 | [-0.14, 0.29] | .01 | [-.03, .04] | .08 |  |  |
| Total BIS | -0.00 | [-0.01, 0.00] | -0.01 | [-0.23, 0.21] | .00 | [-.00, .00] | .01 |  |  |
|  |  |  |  |  |  |  |  | *R^2^*  = .006 | Δ*R^2^*  = .000 |
|  |  |  |  |  |  |  |  | 95% CI[.00,.05] | 95% CI[-.00, .00] |
|  |  |  |  |  |  |  |  |  |  |
| (Intercept) | -0.07 | [-0.39, 0.25] |  |  |  |  |  |  |  |
| MMI Score | 0.02 | [-0.02, 0.05] | 0.10 | [-0.12, 0.32] | .01 | [-.03, .05] | .08 |  |  |
| Total BIS | -0.00 | [-0.01, 0.00] | -0.04 | [-0.27, 0.18] | .00 | [-.02, .02] | .01 |  |  |
| MPI Score | 0.00 | [-0.00, 0.01] | 0.12 | [-0.10, 0.35] | .01 | [-.03, .06] | .10 |  |  |
|  |  |  |  |  |  |  |  | *R^2^*  = .020 | Δ*R^2^*  = .014 |
|  |  |  |  |  |  |  |  | 95% CI[.00,.08] | 95% CI[-.03, .06] |
|  |  |  |  |  |  |  |  |  |  |

*Note.* A significant *b*-weight indicates the beta-weight and semi-partial correlation are also significant. *b* represents unstandardized regression weights. *beta* indicates the standardized regression weights. *sr^2^* represents the semi-partial correlation squared. *r* represents the zero-order correlation. *LL* and *UL* indicate the lower and upper limits of a confidence interval, respectively.
* indicates *p* < .05. ** indicates *p* < .01.
