# Supplementary material for: Effects of media multitasking frequency on a novel volitional multitasking paradigm
Source: PeerJ. 2022 Jan 27;10:e12603. doi: 10.7717/peerj.12603 (PMC8801180; doi:10.7717/peerj.12603)
Supplement: Supplemental Information 6 — Note. A significant b-weight indicates the beta-weight and semi-partial correlation are also significant. b represents unstandardized regression weights. beta indicates the standardized regression weights. sr2 represents the semi-partial correlation squared. r represents the zero-order correlation. LL and UL indicate the lower and upper limits of a confidence interval, respectively. * indicates p < .05. ** indicates p < .01. [file peerj-10-12603-s006.docx]

Supplemental Table S5

*Regression results using Popup_select_ as the criterion*

| Predictor | *b* | *b*  95% CI  [LL, UL] | *beta* | *beta*  95% CI  [LL, UL] | *sr^2^* | *sr^2^*  95% CI  [LL, UL] | *r* | Fit | Difference |
| --- | --- | --- | --- | --- | --- | --- | --- | --- | --- |
| (Intercept) | 1.42** | [1.20, 1.64] |  |  |  |  |  |  |  |
| MMI Score | -0.04 | [-0.11, 0.03] | -0.12 | [-0.37, 0.12] | .02 | [.00, .12] | -.12 |  |  |
|  |  |  |  |  |  |  |  | *R^2^*  = .015 |  |
|  |  |  |  |  |  |  |  | 95% CI[.00,.12] |  |
|  |  |  |  |  |  |  |  |  |  |
| (Intercept) | 1.63** | [1.18, 2.07] |  |  |  |  |  |  |  |
| MMI Score | -0.03 | [-0.10, 0.04] | -0.10 | [-0.35, 0.14] | .01 | [-.04, .06] | -.12 |  |  |
| Attentional | -0.01 | [-0.04, 0.01] | -0.13 | [-0.38, 0.11] | .02 | [-.04, .08] | -.15 |  |  |
|  |  |  |  |  |  |  |  | *R^2^*  = .033 | Δ*R^2^*  = .018 |
|  |  |  |  |  |  |  |  | 95% CI[.00,.13] | 95% CI[-.04, .08] |
|  |  |  |  |  |  |  |  |  |  |
| (Intercept) | 1.71** | [1.22, 2.21] |  |  |  |  |  |  |  |
| MMI Score | -0.03 | [-0.11, 0.04] | -0.12 | [-0.37, 0.13] | .01 | [-.04, .07] | -.12 |  |  |
| Attentional | -0.01 | [-0.03, 0.02] | -0.10 | [-0.36, 0.16] | .01 | [-.04, .05] | -.15 |  |  |
| MPI Score | -0.00 | [-0.01, 0.01] | -0.10 | [-0.36, 0.16] | .01 | [-.04, .05] | -.12 |  |  |
|  |  |  |  |  |  |  |  | *R^2^*  = .042 | Δ*R^2^*  = .009 |
|  |  |  |  |  |  |  |  | 95% CI[.00,.13] | 95% CI[-.04, .05] |
|  |  |  |  |  |  |  |  |  |  |

*Note.* A significant *b*-weight indicates the beta-weight and semi-partial correlation are also significant. *b* represents unstandardized regression weights. *beta* indicates the standardized regression weights. *sr^2^* represents the semi-partial correlation squared. *r* represents the zero-order correlation. *LL* and *UL* indicate the lower and upper limits of a confidence interval, respectively.
* indicates *p* < .05. ** indicates *p* < .01.
