# Supplementary material for: Effects of media multitasking frequency on a novel volitional multitasking paradigm
Source: PeerJ. 2022 Jan 27;10:e12603. doi: 10.7717/peerj.12603 (PMC8801180; doi:10.7717/peerj.12603)
Supplement: Supplemental Information 7 — Primaryreturn refers to average RT on primary task responses following a task switch. Popupignore refers to the average primary task RT on trials where a pop-up was presented, but the secondary task wasn’t chosen. Primarynopopup refers to the average response time on non-popup trials. Primary RT refers to the average response time on all primary task trials. Secondary RT refers to the average response time on all secondary task trials. Primaryrepeat refers to the average RT on primary task trials in which the participant completed the primary task on the preceding trial. [file peerj-10-12603-s007.docx]

*Supplemental Table S6.* Descriptive statistics for the exploratory behavioral measures analyzed.

|  | Primary_return_ | Popup_ignore_ | | Primary_nopopup_ | Primary RT | Secondary RT | Primary_repeat_ |
| --- | --- | --- | --- | --- | --- | --- | --- |
| Mean | 2.69s | 2.33s | 2.31s | | 2.32s | 2.62s | 2.7s |
| SD | 0.71s | 0.46s | 0.42s | | 0.71s | 0.5s | 0.41s |
