# Supplementary material for: Effects of media multitasking frequency on a novel volitional multitasking paradigm
Source: PeerJ. 2022 Jan 27;10:e12603. doi: 10.7717/peerj.12603 (PMC8801180; doi:10.7717/peerj.12603)
Supplement: Supplemental Information 8 — Note. A significant b-weight indicates the beta-weight and semi-partial correlation are also significant. b represents unstandardized regression weights. beta indicates the standardized regression weights. sr2 represents the semi-partial correlation squared. r represents the zero-order correlation. LL and UL indicate the lower and upper limits of a confidence interval, respectively. * indicates p < .05. ** indicates p < .01. [file peerj-10-12603-s008.docx]

Supplemental Table S7

*Regression results using Primary_return_ as the criterion*

| Predictor | *b* | *b*  95% CI  [LL, UL] | *beta* | *beta*  95% CI  [LL, UL] | *sr^2^* | *sr^2^*  95% CI  [LL, UL] | *r* | Fit | Difference |
| --- | --- | --- | --- | --- | --- | --- | --- | --- | --- |
| (Intercept) | 2.27** | [1.82, 2.72] |  |  |  |  |  |  |  |
| MMI Score | 0.15* | [0.00, 0.29] | 0.25 | [0.00, 0.49] | .06 | [.00, .20] | .25* |  |  |
|  |  |  |  |  |  |  |  | *R^2^*  = .061* |  |
|  |  |  |  |  |  |  |  | 95% CI[.00,.20] |  |
|  |  |  |  |  |  |  |  |  |  |
| (Intercept) | 2.45** | [1.29, 3.62] |  |  |  |  |  |  |  |
| MMI Score | 0.15* | [0.00, 0.30] | 0.26 | [0.00, 0.51] | .06 | [-.05, .18] | .25* |  |  |
| Total BIS | -0.00 | [-0.02, 0.02] | -0.04 | [-0.30, 0.21] | .00 | [-.02, .02] | .03 |  |  |
|  |  |  |  |  |  |  |  | *R^2^*  = .062 | Δ*R^2^*  = .002 |
|  |  |  |  |  |  |  |  | 95% CI[.00,.18] | 95% CI[-.02, .02] |
|  |  |  |  |  |  |  |  |  |  |
| (Intercept) | 2.40** | [1.16, 3.64] |  |  |  |  |  |  |  |
| MMI Score | 0.16* | [0.00, 0.31] | 0.26 | [0.00, 0.52] | .06 | [-.05, .18] | .25* |  |  |
| Total BIS | -0.00 | [-0.02, 0.02] | -0.05 | [-0.32, 0.21] | .00 | [-.02, .03] | .03 |  |  |
| MPI Score | 0.00 | [-0.01, 0.02] | 0.04 | [-0.22, 0.29] | .00 | [-.02, .02] | .01 |  |  |
|  |  |  |  |  |  |  |  | *R^2^*  = .064 | Δ*R^2^*  = .001 |
|  |  |  |  |  |  |  |  | 95% CI[.00,.17] | 95% CI[-.02, .02] |
|  |  |  |  |  |  |  |  |  |  |

*Note.* A significant *b*-weight indicates the beta-weight and semi-partial correlation are also significant. *b* represents unstandardized regression weights. *beta* indicates the standardized regression weights. *sr^2^* represents the semi-partial correlation squared. *r* represents the zero-order correlation. *LL* and *UL* indicate the lower and upper limits of a confidence interval, respectively.
* indicates *p* < .05. ** indicates *p* < .01.
