# Supplementary material for: Effects of media multitasking frequency on a novel volitional multitasking paradigm
Source: PeerJ. 2022 Jan 27;10:e12603. doi: 10.7717/peerj.12603 (PMC8801180; doi:10.7717/peerj.12603)
Supplement: Supplemental Information 9 — Note. A significant b-weight indicates the beta-weight and semi-partial correlation are also significant. b represents unstandardized regression weights. beta indicates the standardized regression weights. sr2 represents the semi-partial correlation squared. r represents the zero-order correlation. LL and UL indicate the lower and upper limits of a confidence interval, respectively. * indicates p < .05. ** indicates p < .01. [file peerj-10-12603-s009.docx]

Supplemental Table S8

*Regression results using Popup_ignore_ as the criterion*

| Predictor | *b* | *b*  95% CI  [LL, UL] | *beta* | *beta*  95% CI  [LL, UL] | *sr^2^* | *sr^2^*  95% CI  [LL, UL] | *r* | Fit | Difference |
| --- | --- | --- | --- | --- | --- | --- | --- | --- | --- |
| (Intercept) | 2.06** | [1.83, 2.29] |  |  |  |  |  |  |  |
| MMI Score | 0.09* | [0.02, 0.16] | 0.26 | [0.05, 0.46] | .07 | [.00, .18] | .26* |  |  |
|  |  |  |  |  |  |  |  | *R^2^*  = .065* |  |
|  |  |  |  |  |  |  |  | 95% CI[.00,.18] |  |
|  |  |  |  |  |  |  |  |  |  |
| (Intercept) | 1.89** | [1.27, 2.51] |  |  |  |  |  |  |  |
| MMI Score | 0.09* | [0.01, 0.16] | 0.24 | [0.03, 0.45] | .06 | [-.04, .15] | .26* |  |  |
| TotalcBIS | 0.00 | [-0.01, 0.01] | 0.06 | [-0.15, 0.27] | .00 | [-.02, .03] | .11 |  |  |
|  |  |  |  |  |  |  |  | *R^2^*  = .069* | Δ*R^2^*  = .004 |
|  |  |  |  |  |  |  |  | 95% CI[.00,.18] | 95% CI[-.02, .03] |
|  |  |  |  |  |  |  |  |  |  |
| (Intercept) | 1.66** | [1.02, 2.29] |  |  |  |  |  |  |  |
| MMI Score | 0.10** | [0.03, 0.17] | 0.28 | [0.08, 0.49] | .08 | [-.03, .18] | .26* |  |  |
| Total BIS | -0.00 | [-0.01, 0.01] | -0.01 | [-0.23, 0.20] | .00 | [-.01, .01] | .11 |  |  |
| MPI Score | 0.01* | [0.00, 0.02] | 0.26 | [0.05, 0.47] | .06 | [-.03, .16] | .23* |  |  |
|  |  |  |  |  |  |  |  | *R^2^*  = .131** | Δ*R^2^*  = .062* |
|  |  |  |  |  |  |  |  | 95% CI[.01,.24] | 95% CI[-.03, .16] |
|  |  |  |  |  |  |  |  |  |  |

*Note.* A significant *b*-weight indicates the beta-weight and semi-partial correlation are also significant. *b* represents unstandardized regression weights. *beta* indicates the standardized regression weights. *sr^2^* represents the semi-partial correlation squared. *r* represents the zero-order correlation. *LL* and *UL* indicate the lower and upper limits of a confidence interval, respectively.
* indicates *p* < .05. ** indicates *p* < .01.
