# Supplementary material for: Effects of media multitasking frequency on a novel volitional multitasking paradigm
Source: PeerJ. 2022 Jan 27;10:e12603. doi: 10.7717/peerj.12603 (PMC8801180; doi:10.7717/peerj.12603)
Supplement: Supplemental Information 10 — Note. A significant b-weight indicates the beta-weight and semi-partial correlation are also significant. b represents unstandardized regression weights. beta indicates the standardized regression weights. sr2 represents the semi-partial correlation squared. r represents the zero-order correlation. LL and UL indicate the lower and upper limits of a confidence interval, respectively. * indicates p < .05. ** indicates p < .01. [file peerj-10-12603-s010.docx]

Supplemental Table S9

*Regression results using Primary_nopopup_ as the criterion*

| Predictor | *b* | *b*  95% CI  [LL, UL] | *beta* | *beta*  95% CI  [LL, UL] | *sr^2^* | *sr^2^*  95% CI  [LL, UL] | *r* | Fit | Difference |
| --- | --- | --- | --- | --- | --- | --- | --- | --- | --- |
| (Intercept) | 2.08** | [1.87, 2.30] |  |  |  |  |  |  |  |
| MMI Score | 0.08* | [0.01, 0.14] | 0.24 | [0.03, 0.44] | .06 | [.00, .17] | .24* |  |  |
|  |  |  |  |  |  |  |  | *R^2^*  = .057* |  |
|  |  |  |  |  |  |  |  | 95% CI[.00,.17] |  |
|  |  |  |  |  |  |  |  |  |  |
| (Intercept) | 1.90** | [1.33, 2.47] |  |  |  |  |  |  |  |
| MMI Score | 0.07* | [0.00, 0.14] | 0.22 | [0.01, 0.43] | .05 | [-.04, .13] | .24* |  |  |
| Total BIS | 0.00 | [-0.01, 0.01] | 0.07 | [-0.14, 0.28] | .01 | [-.02, .03] | .12 |  |  |
|  |  |  |  |  |  |  |  | *R^2^*  = .062 | Δ*R^2^*  = .005 |
|  |  |  |  |  |  |  |  | 95% CI[.00,.17] | 95% CI[-.02, .03] |
|  |  |  |  |  |  |  |  |  |  |
| (Intercept) | 1.72** | [1.13, 2.30] |  |  |  |  |  |  |  |
| MMI Score | 0.08* | [0.02, 0.15] | 0.26 | [0.05, 0.47] | .06 | [-.03, .16] | .24* |  |  |
| Total BIS | 0.00 | [-0.01, 0.01] | 0.01 | [-0.20, 0.23] | .00 | [-.00, .01] | .12 |  |  |
| MPI Score | 0.01* | [0.00, 0.02] | 0.21 | [0.00, 0.43] | .04 | [-.04, .12] | .19 |  |  |
|  |  |  |  |  |  |  |  | *R^2^*  = .104* | Δ*R^2^*  = .042* |
|  |  |  |  |  |  |  |  | 95% CI[.00,.21] | 95% CI[-.04, .12] |
|  |  |  |  |  |  |  |  |  |  |

*Note.* A significant *b*-weight indicates the beta-weight and semi-partial correlation are also significant. *b* represents unstandardized regression weights. *beta* indicates the standardized regression weights. *sr^2^* represents the semi-partial correlation squared. *r* represents the zero-order correlation. *LL* and *UL* indicate the lower and upper limits of a confidence interval, respectively.
* indicates *p* < .05. ** indicates *p* < .01.
