# Supplementary material for: Effects of media multitasking frequency on a novel volitional multitasking paradigm
Source: PeerJ. 2022 Jan 27;10:e12603. doi: 10.7717/peerj.12603 (PMC8801180; doi:10.7717/peerj.12603)
Supplement: Supplemental Information 12 — Note. A significant b-weight indicates the beta-weight and semi-partial correlation are also significant. b represents unstandardized regression weights. beta indicates the standardized regression weights. sr2 represents the semi-partial correlation squared. r represents the zero-order correlation. LL and UL indicate the lower and upper limits of a confidence interval, respectively. * indicates p < .05. ** indicates p < .01. [file peerj-10-12603-s012.docx]

Supplemental Table S11

*Regression results using Secondary RT as the criterion*

| Predictor | *b* | *b*  95% CI  [LL, UL] | *beta* | *beta*  95% CI  [LL, UL] | *sr^2^* | *sr^2^*  95% CI  [LL, UL] | *r* | Fit | Difference |
| --- | --- | --- | --- | --- | --- | --- | --- | --- | --- |
| (Intercept) | 2.51** | [2.19, 2.83] |  |  |  |  |  |  |  |
| MMI Score | 0.04 | [-0.06, 0.14] | 0.09 | [-0.16, 0.35] | .01 | [.00, .10] | .09 |  |  |
|  |  |  |  |  |  |  |  | *R^2^*  = .009 |  |
|  |  |  |  |  |  |  |  | 95% CI[.00,.10] |  |
|  |  |  |  |  |  |  |  |  |  |
| (Intercept) | 3.07** | [2.24, 3.90] |  |  |  |  |  |  |  |
| MMI Score | 0.06 | [-0.05, 0.16] | 0.14 | [-0.12, 0.40] | .02 | [-.05, .08] | .09 |  |  |
| Total BIS | -0.01 | [-0.02, 0.00] | -0.19 | [-0.45, 0.07] | .03 | [-.05, .12] | -.16 |  |  |
|  |  |  |  |  |  |  |  | *R^2^*  = .043 | Δ*R^2^*  = .034 |
|  |  |  |  |  |  |  |  | 95% CI[.00,.15] | 95% CI[-.05, .12] |
|  |  |  |  |  |  |  |  |  |  |
| (Intercept) | 3.08** | [2.20, 3.96] |  |  |  |  |  |  |  |
| MMI Score | 0.06 | [-0.05, 0.16] | 0.14 | [-0.12, 0.40] | .02 | [-.05, .08] | .09 |  |  |
| Total BIS | -0.01 | [-0.02, 0.00] | -0.19 | [-0.46, 0.08] | .03 | [-.05, .11] | -.16 |  |  |
| MPI Score | -0.00 | [-0.01, 0.01] | -0.01 | [-0.27, 0.26] | .00 | [-.00, .00] | -.07 |  |  |
|  |  |  |  |  |  |  |  | *R^2^*  = .043 | Δ*R^2^*  = .000 |
|  |  |  |  |  |  |  |  | 95% CI[.00,.14] | 95% CI[-.00, .00] |
|  |  |  |  |  |  |  |  |  |  |

*Note.* A significant *b*-weight indicates the beta-weight and semi-partial correlation are also significant. *b* represents unstandardized regression weights. *beta* indicates the standardized regression weights. *sr^2^* represents the semi-partial correlation squared. *r* represents the zero-order correlation. *LL* and *UL* indicate the lower and upper limits of a confidence interval, respectively.
* indicates *p* < .05. ** indicates *p* < .01.
